# Retinal versus physical stimulus size as determinants of visual perception in simultanagnosia

**DOI:** 10.1016/j.neuropsychologia.2010.02.013

**Published:** 2010-05

**Authors:** Elisabeth Huberle, Jon Driver, Hans-Otto Karnath

**Affiliations:** a Section of Neuropsychology, Center of Neurology, Hertie-Institute for Clinical Brain Research, University of Tübingen, Tübingen, Germany; b Department of General Neurology, Center of Neurology, Hertie-Institute for Clinical Brain Research, University of Tübingen, Tübingen, Germany; c UCL Institute of Cognitive Neuroscience, University College London, London, UK

**Keywords:** Simultanagnosia, Visual perception, Spatial attention, Retinal size, Global/local

## Abstract

Patients with simultanagnosia following bilateral parieto-temporo-occipital brain damage show a characteristic impairment of global gestalt perception, while their perception of individual objects or elements remains intact. For instance, when shown ‘hierarchical’ stimuli comprising a larger global object (e.g. a large letter) made up from smaller components (e.g. multiple small letters), they typically report seeing one of the smaller components but not the global figure. Recent work on simultanagnosia revealed that global perception can be improved if local element spacing is reduced. However, it is still unclear whether the retinal separation or the physical (post-size-constancy) spatial separation is critical. Here, we presented various hierarchical global/local letter stimuli at different viewing distances and sizes to separate the impacts of retinal versus physical size. Our findings indicate a key role for visual angle in determining simultanagnosic perception. We observed that not only retinal spacing (in terms of visual angle) between local elements had a major impact on global perception in simultanagnosia, but also the physical size of the separation between local elements, provided that binocular cues to viewing distance were available. The results indicate both pre-size-constancy retinal influences and binocular-post-constancy influences upon conscious perception in simultanagnosia.

## 1 Introduction

Efficient visual processing of the environment requires the perception of multiple aspects ranging from local details of individual objects to more holistic representation of the global scene. Separate brain mechanisms may process these different aspects. Patients with bilateral parieto-temporo-occipital brain damage exhibit a severe deficit in global scene perception, while their recognition of individual objects or elements remains intact (Bálint, 1909; Luria, 1959; Wolpert, 1924). This disorder has been termed ‘simultanagnosia’ (Bálint, 1909; Friedman-Hill, Robertson, & Treisman, 1995; Karnath, 2006; Rafal, 1997; Rizzo & Hurtig, 1987). Patients with simultanagnosia show a ‘piecemeal’ perception of their visual surroundings and are typically not able to perceive more than one object at a time (Luria, 1959). Although this clinical presentation is well known, the mechanisms underlying simultanagnosia remain under debate. Several authors suggested impaired working memory processes for spatial locations (Coslett & Saffran, 1991; Friedman & Goldman-Rakic, 1994; Friedman-Hill et al., 1995; for review see Ungerleider, Courtney, & Haxby, 1998), others emphasized possible roles for sustained attention (Rizzo & Robin, 1990) or visuo-spatial processes (Duncan et al., 2003; Huberle & Karnath, 2006; Kinsbourne & Warrington, 1962) required for the efficient integration of visual information (Friedman-Hill et al., 1995).

Several studies of simultanagnosic patients have focused on the disturbed conscious perception of a global gestalt despite preserved awareness for the local elements that together define the global stimulus (e.g. Clavagnier, Fruhmann Berger, Klockgether, Moskau, & Karnath, 2006; Dalrymple, Kingstone, & Barton, 2007; Karnath, Ferber, Rorden, & Driver, 2000; Navon, 1977). Recent findings in two patients with simultanagnosia demonstrated that whether or not the larger gestalt can be reported is modulated by the spatial separation between the individual elements of hierarchical organized complex visual arrays (Huberle & Karnath, 2006). However, in that latter study, the physical separations covaried with the retinal stimulus sizes. It has long been known from classic psychophysical studies of normal vision (e.g. Emmert, 1881) that the perceived size of a visual object or element can remain fairly constant independent of its current retinal size, as when viewing the same object from difference distances (e.g. Fitzpatrick, Pasnak, & Tyer, 1982). Even infants at the age of four months might react rather to the physical than the retinal size of seen objects (Granrud, 2006).

The present study was designed to contrast global gestalt perception evoked by changes in the physical size versus changes in the retinal size of visual elements for hierarchical Navon-like letters in a patient exhibiting the hallmark symptoms of simultanagnosia. We presented stimuli at various distances to the patient while holding the physical size of the visual stimuli constant in critical conditions. These results were compared against situations in which the retinal size of the stimuli was kept constant. Finally, given that (in normals) perceived visual size-constancy can depend on the availability of depth cues, including those from binocular disparity (e.g. Bradshaw, Parton, & Eagle, 1998; Jackson, Newport, & Shaw, 2002; Subramanian & Dickinson, 2004), we also varied whether disparity information was available to our patient or not.

## 2 Materials and methods

### 2.1 Patient HW

**Fig. 1:**
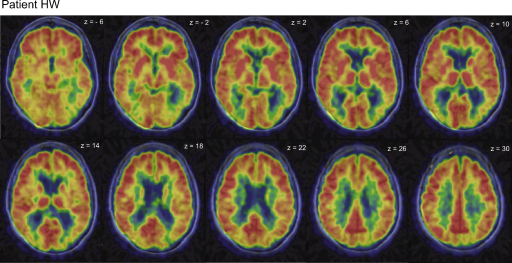
18-Fluorodeoxyglucose positron emission tomography (FDG-PET) scans overlayed with the anatomical magnetic resonance imaging (MRI) scans for patient HW reveal reduced metabolism in the parieto-temporo-occipital cortex bilaterally; see main text.

HW, a 71-year-old, right-handed woman, was admitted to our department with a history of (reportedly) unspecific progressive ‘visual impairment’ for several years affecting visual orienting, reading abilities and other daily activities such as counting coins, descending stairs and cooking. Standard neurological examination was normal. Visual fields were intact (investigated by standard Goldmann-Perimetry) as well as the function of all other cranial nerves. T1- and T2-weighted magnetic resonance imaging of HW showed no obvious pathological results. However, 18-fluorodeoxyglucose positron emission tomography (FDG-PET) revealed reduced metabolism in the parieto-temporo-occipital cortex bilaterally (Fig. 1), leading to the diagnosis of posterior cortical atrophy (PCA). In accordance with recent findings (Tang-Wai et al., 2004), cerebrospinal fluid analysis indicated a variant of Alzheimer's disease. Ophthalmologic examination showed reduced visual acuity of the right eye (near 0.5/far 0.6) in the context of early-stage glaucoma, while normal results were obtained for the left eye (near 0.9/far 0.9).

Neuropsychological testing revealed severe visual simultanagnosia. The patient was not able to identify the large letter at the global scale for any of 10 Navon hierarchical letter stimuli (Navon, 1977), while recognition of the letters at the local scale (that together made up the larger global letter) was always intact. In accordance with recent findings from other simultanagnosic patients (Huberle & Karnath, 2006), HW showed increased performance for global shape recognition when the composite stimulus had smaller inter-element distances at the local level. Suitable with the diagnosis of simultanagnosia, HW also was unable to report the general context for complex images such as the Broken Window Picture from the Stanford Binet Intelligence Test (Binet & Simon, 1905; Roid, 2003). In addition to simultanagnosia, HW showed (possibly related) signs of constructive apraxia that prevented her from copying visual objects of increasing complexity. No signs of spatial neglect, visual agnosia or visual field defects were observed.

### 2.2 Visual stimuli, design and presentation procedures for the three experiments

**Fig. 2:**
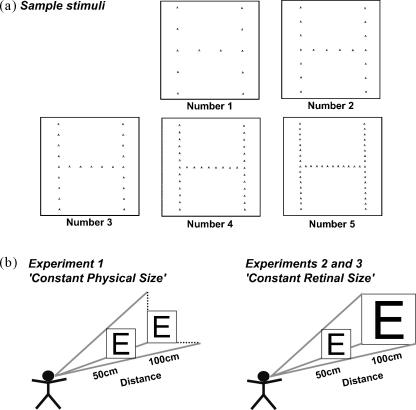
Navon-like hierarchical letter stimuli, comprising a larger ‘global’ letter constructed from multiple repeated smaller ‘local’ letters, were used in all experiments. Five different letter identities could appear at the global or repeated local level (A, B, E, H, N), with the global letter identity always differing from the local (a). The spacing between letters at the local scale varied parametrically across five conditions (schematically labelled as ‘Numbers 1 to 5’) by adding local letters while keeping the size of the global letter unchanged (b). The stimuli were presented at two different viewing distances (‘close’ or ‘far’) between the patient's eyes and the presentation monitor. We either kept the physical displays themselves constant (Experiment 1, ‘Constant Physical Size’) while varying viewing distance; or else adjusted their physical size for the far viewing distance such that the retinal size of the stimuli then remained identical across difference viewing distances (Experiments 2 and 3, ‘Constant Retinal Size’).

Navon hierarchical letter stimuli (Navon, 1977), each comprising a large letter (global scale) constructed from multiple smaller letters (local scale) were used in all experiments (see Fig. 2a for examples). Five different letter identities (A, B, E, H, or N) were used at the global and local scale. All possible combinations of incongruent stimuli (i.e. different letter identities at the global and local scale) were utilized, resulting in 20 different combinations. All 20 were re-generated at five different inter-element distances between the letters at the local scale (referred to schematically here as numbers 1 to 5; Fig. 2a) by varying the actual number of local elements.

Prior to all experiments, HW was familiarized with the stimuli. Each trial was then initiated by the experimenter when the patient indicated readiness. All stimuli were presented in a random order that counterbalanced for the letter at the global scale, the letter at the local scale and the visual angle between the letters at the local scale (see below). After a delay of 600 ms, the stimulus appeared at the center of a computer monitor for a presentation duration of 5000 ms. HW was instructed to identify the letter at the global scale, while the experimenter coded her verbal responses. All stimuli were presented on the monitor in an otherwise completely darkened room. The outline of the monitor was occluded in black with a square opening, in which the stimuli were centrally presented. The experiments were conducted in two blocks over a course of four months. To avoid any interactions between experimental results and the progression of PCA, we avoided direct comparison between the results of the different experiments. Instead, we used all conditions for the close viewing distance – which were identical across all experiments – as a reference or baseline.

#### 2.2.1 Experiment 1

In our initial study (*‘Constant Physical Size’* Experiment), we employed Navon hierarchical letter stimuli with five different visual angles between neighboring letters at the local scale, while the global size remained unchanged across conditions. In detail, the smaller the visual angle, the more letters at the local scale were presented. These stimuli were equivalent to those applied in another recent study from our laboratory on patients with simultanagnosia (Huberle & Karnath, 2006), except that here we used white stimuli on a black background rather than vice-versa (to minimize the light presented in the dark testing room). But in addition, we now also manipulated the viewing distance between the patient's eyes and the monitor on which the stimuli were presented (see Fig. 2b). The physical size of the stimuli remained unchanged, only the viewing distance (and thereby the retinal size) was manipulated. We used a ‘close’ viewing distance of 50 cm and a ‘far’ viewing distance of 100 cm. At the close distance, the global letter covered retinal visual angles of 10.9° × 10.9°, the local letter were 0.35° × 0.35° each, with the following visual angles separating adjacent local letters: 2.55° (Number 1), 1.70° (Number 2), 1.28° (Number 3), 0.85° (Number 4), and 0.64° (Number 5). For the far distance, the same external visual stimuli resulted in the following retinal visual angles for the same stimulus set: the global letter covered 5.46° × 5.46°, while each local letter was 0.18° × 0.18°. The possible separations between adjacent local letters at the far viewing distance resulted in the following retinal sizes: 1.28° (spacing Number 1), 0.85° (Number 2), 0.64° (Number 3), 0.43° (Number 4), and 0.32° (Number 5). As a result of this procedure, the final stimulus set consisted of 100 displays (five possible letters at the global scale each constructed from any one of four repeated letters at the local scale, presented at five different inter-element spacings) that were presented once (100 trials) at each of the two viewing distances (200 trials in total), starting with the close viewing distance.

#### 2.2.2 Experiments 2 and 3

In a second (*‘Constant Retinal Size – Binocular’* Experiment) and third (*‘Constant Retinal Size – Monocular’* Experiment*)* experiment, we used the same set of displays as for Experiment 1 at the close viewing distance, but now changed the stimuli for the far viewing distance and enlarged those displays by a factor of two (see Fig. 2b). Due to this enlargement, the retinal size of the stimuli remained constant, while the physical size now varied across the two viewing distances instead. This procedure resulted in the following stimulus parameters: the global letter had a retinal size of 10.9° × 10.9° and each local letter 0.35° × 0.35°, while the retinal separation between adjacent local letters was 2.55° (spacing Number 1), 1.70° (Number 2), 1.28° (Number 3), 0.85° (Number 4), and 0.64° (Number 5). In addition, the physical size of the square window on the presentation monitor was adjusted by a factor of two for the far viewing distance. A total number of 100 stimuli was again used at each viewing distance, which were presented twice resulting in a total number of 400 trials. The experiment was conducted in two blocks. While in the first block the close viewing distance was tested first, in the second block, the opposite order was used.

In Experiment 3, the same set of stimuli was presented as for Experiment 2, but HW's perception was now restricted to monocular vision by patching the patient's right eye. This was done to examine a possible impact of binocular-disparity cues.

## 3 Results

### 3.1 Experiment 1 (‘Constant Physical Size’ Experiment)

**Fig. 3:**
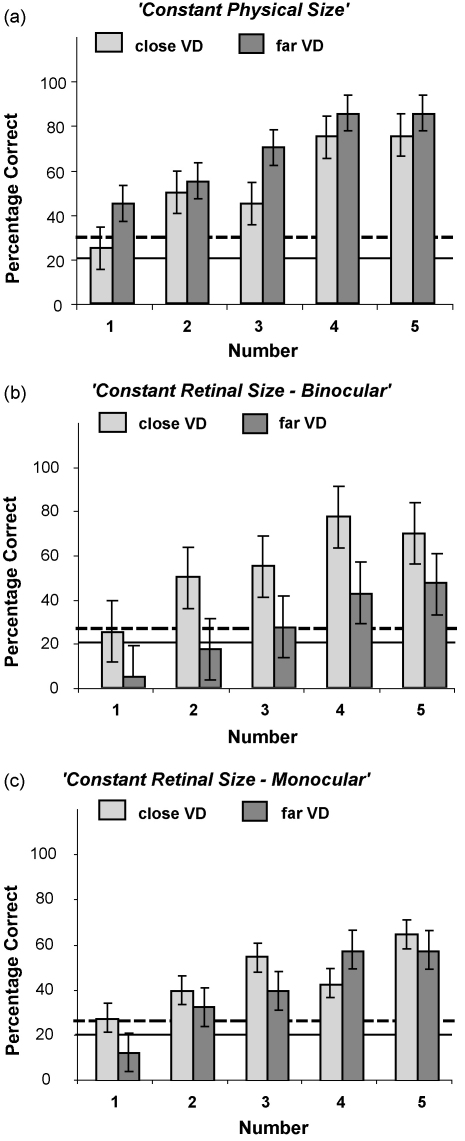
Average percent (with standard errors shown) of correct global-letter report for patient HW in the sub-conditions of (a) Experiment 1, (b) Experiments 2, and (c) Experiment 3. The solid horizontal line in each graph indicates chance-level performance; the dashed lines indicate the binomial 95%-confidence interval for above-chance performance.

HW's severely impaired global gestalt perception for Navon hierarchical letter stimuli improved to above-chance levels for decreasing inter-element spacing (see Fig. 3a). This aspect of the new results replicates our previous observations from two other patients with simultanagnosia (Huberle & Karnath, 2006). The impact of inter-element spacing was found here for both viewing distances (for the close viewing distance: *χ*^2^(1) = 14.65, *p* < 0.01; for the far: *χ*^2^(1) = 11.77, *p* < 0.05). In addition, HW's performance in reporting the global letter was significantly better overall for the far viewing distance compared to the close viewing distance (McNemar Test: *p* < 0.05). Since the physical size of the stimulus displays was equivalent across the two viewing distances, presumably the changed retinal size is responsible for the impact of viewing distance.

### 3.2 Experiment 2 (‘Constant Retinal Size – Binocular’ Experiment)

HW's performance in the close viewing distance was similar to Experiment 1 and improved for reduced inter-element spacing at both viewing distances (close viewing distance: *χ*^2^(1) = 26.80, *p* < 0.001; far viewing distance, *χ*^2^(1) = 24.41, *p* < 0.001) (Fig. 3b). Although the retinal size now remained constant at the close and far viewing distance, performance was better overall for the close compared to the far viewing distance (McNemar Test: *p* < 0.001). This particular aspect of our results cannot reflect retinal factors, so presumably reflects some influence of physical stimulus size/spacing, as may be perceived via size-constancy in the presence of distance cues such as binocular disparity. If indeed this particular effect does rely on disparity information, it should disappear under monocular viewing as tested in our final experiment.

### 3.3 Experiment 3 (‘Constant Retinal Size – Monocular’ Experiment)

Under the monocular viewing condition, HW's performance in the close viewing distance was similar to Experiment 1 and improved for reduced inter-element spacing at both viewing distances (close viewing distance: *χ*^2^(1) = 13.41, *p* < 0.01; far viewing distance: *χ*^2^(1) = 23.75, *p* < 0.001; see Fig. 3c). But a critical change was that, unlike in Experiment 2, there was no longer any difference in performance between the close and the far viewing distance (McNemar Test: *p* = 0.14). This difference between Experiments 2 and 3 indicates that the effect of physical stimulus size, found in Experiment 2 only, depends on binocular vision, presumably reflecting the role of disparity cues to depth in allowing size-constancy (and thus perception of physical rather than retinal size) to emerge when binocular vision is allowed, unlike the monocular conditions of Experiment 3.

## 4 Discussion

Patients with simultanagnosia are typically severely impaired at reporting global aspects of scenes or stimuli comprising multiple elements, while recognition of the single objects or local details is preserved. This is exemplified by their performance with Navon-like global/local hierarchical letters (Clavagnier et al., 2006; Karnath et al., 2000; Rafal & Robertson, 1995), for which simultanagnosic patients can typically report the local letters, but struggle to identify the larger global letter that is made up from the local letters.

A recent study (Huberle & Karnath, 2006) observed that global perception of Navon-like hierarchical letters can be improved when the spacing of the local elements is reduced (see also McCrea, Buxbaum, & Coslett, 2006). This raises the question, that was studied in the present study, whether the critical spacing factor for determining simultanagnosic perception concerns the retinal separation of the local elements in terms of visual angles, or instead the physical size of the spacing, as might be encoded in post-size-constancy visual representations (e.g. Emmert, 1881; Fitzpatrick et al., 1982).

Here we presented Navon-like hierarchical letter stimuli with different inter-element spacings at the local level (as in Huberle & Karnath, 2006), but critically also at different viewing distances, either while holding the physical stimulus sizes constant so that retinal visual angles covaried with distance (Experiment 1); or else changing physical stimulus size at the different viewing distances so that the retinal visual angles remained the same across those distances (Experiments 2 and 3). We further manipulated whether viewing was binocular or monocular.

In accord with findings in other simultanagnosic patients (Huberle & Karnath, 2006; McCrea et al., 2006), we found here that patient HW's perception of the global letter improved as the spacing of the component local letters was reduced. But our present results go beyond those previous findings. Patient HW's global performance was better for the far than the near viewing distance (see Fig. 3a). Since the *physical* stimulus sizes were equivalent for the two viewing distances in Experiment 1, this outcome must reflect the closer *retinal* spacing of local elements in the far condition of that experiment. On the other hand, Experiment 2 found better performance for the close than the far viewing distance (see Fig. 3b), when using stimuli whose *physical* size was scaled to yield equivalent retinal visual angles at the different viewing distances. This finding likely indicates post-size-constancy influence on simultanagnosic perception (i.e. that not solely retinal factors matter, but also the physical spacing of the stimuli). In addition, it should be noted that the global perception in Experiment 1 showed the tendency to be better for the far than the close viewing distance for identical retinal visual angles between adjacent local letters (1.28°: 45% vs. 45%, 0.85°: 75% vs. 55%, 0.64°: 75% vs. 70%). Apart from the distance between the individual elements, the local letters had smaller retinal visual angles in the far viewing distance. It thus can be speculated that the retinal visual angle of the local letter and possibly also its relation to the global letter as well as the visual angle between adjacent local letters influence global recognition in patients with simultanagnosia. Saliency could be a common concept to better understand these findings. Similar observations were made several decades ago in healthy observers suggesting a critical role of the retinal spacing between the local elements of a complex array (Fox & Mayhew, 1979; Gillam, 1981; Prytulak and Bordie, 1975). Besides a critical role of the distance between adjacent local elements of a complex visual array, other groups have focused on tunnel-like vision in simultanagnosics to explain impaired global processing (for an overview see Farah, 1990). Recent findings (Dalrymple, Bischof, Cameron, Barton, Kingstone, in press) argued that retinal visual angles might be limited to a field of less than 2° × 2° for global objects. However, HW's present performance and the data of earlier patients (Huberle & Karnath, 2006) using the same type of stimuli demonstrated that global perception can reach a degree that exceeds chance level even if the retinal visual angles reach larger sizes (e.g., beyond 11° × 11° as in the present case).

Interestingly, the effect from Experiment 2 was eliminated in Experiment 3, when restricting patient HW to monocular rather than binocular vision. This suggests a binocular source for the impact of the size of physical rather than just retinal stimulus-spacing in Experiment 2. This idea would concur with considerable evidence in healthy observers indicating that size-constancy in visual perception can depend on binocular cues and viewing depth (e.g. Bruggeman, Yonas, & Konczak, 2007; Day, 1972; Pilewski & Martin, 1991; Westheimer, 1972). Binocular-disparity cues are first encoded at rather early stages of cortical visual processing (Barlow, Blakemore, & Pettigrew, 1967; Cumming & Parker, 2000; Holmes, 1945; Hubel & Wiesel, 1962; Poggio & Fischer, 1977; Trotter, Celebrini, Stricanne, Thorpe, & Imbert, 1996; for review see Roe, Parker, Born, & DeAngelis, 2007), which presumably remained functionally intact in patient HW. Binocular cues are thus able to produce some impact of physical stimulus size at different viewing distances, rather than solely of retinal factors even though the latter clearly do impact also on the global recognition performance of HW (see Experiment 1). Further support for intact early visual cortical processes in simultanagnosia came from a simultanagnosic patient showing intact automatic processing of color and size (Demeyere, Rzeskiewicz, Humphreys, & Humphreys, 2008). Also the present results argue against a general deficit for monocular processing. The level of performance for the ‘baseline’ conditions of the close viewing distance was similar under binocular and monocular viewing conditions. Support for an instable global perception has been found in a more general context by several investigations that observed an increased performance to report both targets of a two-unit array if the items had a semantic relation (Coslett & Lie, 2008). Further evidence came from observations in a patient with visual extinction whose interactive perceptual and attentional limits were investigated (Shalev, Chajut, & Humpreys, 2005). The patient's contralesional deficits could be manipulated by changing the saliency of the stimulus and cueing attention with a strong interaction under conditions of high perceptual saliency. In accord, another study indicated that the processing of color and size of a multi-unit array appeared to be largely preserved under conditions of distributed attention (Demeyere et al., 2008). Recent fMRI findings in normals indicated that neural activity can be modulated by the perceived rather than purely retinal size even in primary visual cortex (Murray, Boyaci, & Kersten, 2006). On the other hand, the representation of disparity in V1 alone cannot fully account for depth perception (Bakin, Nakayama, & Gilbert, 2000; Cumming & Parker, 1997, 1999, 2000; Janssen, Vogels, Liu, & O, 2003; Nienborg & Cumming, 2006) with extrastriate areas also contributing (e.g. Hubel & Wiesel, 1970; Thomas, Cumming, & Parker, 2002). Finally, it should be noted that our data argue against a major role of acuity underlying the difference in global recognition performance between the different viewing distances as acuity per se should just lead to a shift of overall performance between Experiments 2 and 3, rather than selectively influencing the distance effects.

In conclusion, the present findings confirm that the spacing of local elements has a significant impact on global perception in simultanagnosia. They further show that not only retinal spacing (in terms of visual angle) between local elements has a major impact on global perception in simultanagnosia, but – above and beyond retinal factors – also the physical size of the separation between local elements provided that binocular cues to viewing distance are available. These findings indicate that simultanagnosia is constrained by both retinal factors and some preserved influence of post-size-constancy representations that emerge due to binocular cues. Preserved processing in the occipital cortex seems a possible source for these influences.
